# Low-volume high-intensity interval training improves cardiometabolic health, work ability and well-being in severely obese individuals: a randomized-controlled trial sub-study

**DOI:** 10.1186/s12967-020-02592-6

**Published:** 2020-11-07

**Authors:** Dejan Reljic, Fabienne Frenk, Hans J. Herrmann, Markus F. Neurath, Yurdagül Zopf

**Affiliations:** 1 Hector-Center for Nutrition, Exercise and Sports, Department of Medicine 1, University Hospital Erlangen, Friedrich-Alexander University Erlangen-Nürnberg, Ulmenweg 18, 91054 Erlangen, Germany; 2 Department of Medicine 1, University Hospital Erlangen, Friedrich-Alexander University Erlangen-Nürnberg, Erlangen, Germany

**Keywords:** Obesity, Metabolic syndrome, High-intensity interval training, Aerobic exercise, Cardiorespiratory fitness, Weight loss, Psychological health, Work ability

## Abstract

**Background:**

Obesity is associated with impaired health and lower work ability. Increased physical activity is a cornerstone in the treatment of obesity and related risk factors. Recently, high-intensity interval training (HIIT) has emerged as a popular exercise option. However, data regarding the effects on cardiometabolic health, perceived work ability and well-being in severely obese individuals are lacking.

**Methods:**

Sixty-five obese individuals with sedentary occupation (48.7 ± 9.9 years, BMI: 39.6 ± 7.1 kg/m^2^) were randomly allocated to an extremely time-efficient HIIT (5 × 1 min at 80–95% maximal heart rate on cycle ergometers, 2×/week for 12 weeks) or an inactive control group (CON). Both groups received nutritional counseling to support weight loss. Primary outcome was maximal oxygen uptake (VO_2max_), secondary outcomes were cardiometabolic risk indices, body composition, work ability index (WAI), quality of life (QoL, EQ-5D-5L-questionnaire) and perceived stress (PSQ-questionnaire).

**Results:**

Mean body weight reduction was 5.3 kg [95% confidence interval (95% CI) − 7.3 to − 3.3 kg] in the HIIT group (P < 0.001) and 3.7 kg (95% CI − 5.3 to − 2.1 kg) in CON (P < 0.001), respectively. Only the HIIT group showed significant (P < 0.001) changes in VO_2max_ [+ 3.5 mL/kg/min (95% CI 2.5 to 4.6 mL/kg/min)], waist circumference [–7.5 cm (95% CI − 9.8 to − 5.1 kg)], mean arterial blood pressure [− 11 mmHg (95% CI − 14 to − 8 mmHg)], WAI [+ 3.0 points (95% CI 1.7 to 4.3 points)] and QoL [+ 10% (95% CI 5 to 16%)]. In CON, none of these parameters improved significantly.

**Conclusions:**

Low-volume HIIT may induce significant improvements in cardiometabolic health, especially VO_2max_, WAI and well-being in obese individuals after only 12 weeks. Our results underpin the wide range of benefits on health and subjective measures through exercise that go well beyond simple weight loss through dietary restriction alone.

**Trial registration:** ClinicalTrials.gov Id: NCT03306069. Registered 10 October 2017, https://clinicaltrials.gov/ct2/show/NCT03306069.

## Background

The prevalence of obesity has shown a drastic rise in almost all parts of the world over the last decades [1]. Obesity is defined as an abnormally high accumulation of body fat that is associated with an increased risk of several chronic diseases [2, 3] and premature mortality [4]. The occurrence of additional cardiometabolic risk factors along with excess body fat, such as hypertension, dyslipidemia or hyperglycemia, further increases the risk of cardiovascular disease (CVD) and mortality [5]. Moreover, it has been shown that obesity is associated with a diminished quality of life (QoL) in the general population [6] and higher rates of absenteeism [7], early retirement [8] and lower work ability [9] among employees. Consequently, obesity is considered a significant public health concern and there is a dire need for effective treatment strategies [2].

Adequate dietary changes and increased physical activity (PA) are crucial components in the treatment of obesity and related comorbidities. The degree of cardiorespiratory fitness (CRF, typically expressed as maximal oxygen uptake, VO_2max_) has been shown to be an independent predictor of CVD and mortality—stronger than other established risk factors, such as smoking, hypertension or diabetes in both normal-weight and overweight/obese individuals [10].

However, many obese individuals do not achieve the recommended 150 min of PA per week [11] and the widespread adoption of a sedentary lifestyle has been pointed out as a major contributor to the increase in the prevalence of obesity [12, 13]. In addition, sedentary behavior has been recognized as an individual risk factor for cardiometabolic disorders [14]. Thus, obese individuals with insufficient leisure-time PA, sedentary occupation (e.g. office workers) and low CRF levels appear to be at particularly high risk for developing serious health conditions [15, 16].

The underlying reasons why obese individuals do not participate in sufficient PA are manifold but—as in the general population—the most commonly cited barrier is “lack of time” [17]. Hence, the development of less time-consuming exercise modalities has recently gained increasing attention [18]. In this context, high‐intensity interval training (HIIT) has emerged as a time-efficient and effective exercise strategy for achieving health‐relevant benefits. HIIT is a type of cardiovascular exercise that involves brief intense bouts of PA separated by recovery periods of low-intensity activity [19]. It has been demonstrated that HIIT can improve CRF and various cardiometabolic risk markers in normal-weight, overweight and moderately obese individuals effectively within only a few weeks [19, 20]. More specifically, a recent meta-analysis of 22 studies, in which various HIIT protocols were applied in overweight and/or moderately obese adults, reported significant improvements in body composition, lipid metabolism and aerobic capacity variables including moderate effect sizes on body fat (standardized mean difference, SMD, = 0.61) and cholesterol levels (SMD = 0.47) and a large effect size on VO_2max_ (SMD = 0.97) [21]. In these studies, HIIT was performed 3–5 times weekly, with an average session duration of ~ 30 min, and over an average period of 10 weeks [21].

Furthermore, there is increasing evidence that HIIT is at least similar effective to traditional moderate-intensity continuous training (MICT) for reducing body fat mass, despite lower time commitment [21–27]. However, to date, far less is known about the efficacy of HIIT to improve health status in severely obese individuals with clustering of cardiometabolic disorders, whose health risks are substantially higher than in overweight or moderately obese individuals [28], and who may face particular challenges to engage in exercise programs due to physical impairments (e.g. joint problems). Moreover, to the best of our knowledge, the impact of HIIT on perceived work ability and well-being in severely obese employees has not yet been investigated.

The aim of the present study was, therefore, to investigate the efficacy of an extremely time-efficient, low-volume HIIT protocol, previously proven effective in improving CRF and other health markers in sedentary, normal-weight individuals [29], on cardiometabolic health and self-reported outcomes in severely obese individuals at increased cardiometabolic risk with sedentary occupation. Based on data obtained from previous research [29], we hypothesized that low-volume HIIT would be effective in improving cardiometabolic health, work ability, QoL and perceived stress in this particular risk group.

## Materials and methods

### Study design

This study was a sub-study of a larger randomized controlled trial, investigating the effects of different interval training protocols on a variety of clinical parameters in obese individuals at increased cardiometabolic risk. The present sub-study focused specifically on the effects of low-volume HIIT on cardiometabolic health, work ability and well-being in a sub-sample of the main trial (i.e. obese employees) and consisted of a 12-week exercise intervention, nutritional counseling and pre-/post-intervention health examinations. Further results of the main study for other outcomes will be reported in the future. Primary outcome of this study was VO_2max_, secondary outcomes were various other cardiometabolic risk markers (waist circumference, blood pressure, blood glucose, cholesterol and triglyceride concentrations), body composition and self-reported work ability, QoL and perceived stress.

All participants were fully informed about the objectives and methods of the study, which conformed to the Helsinki Declaration and provided written consent before participation. The protocol of the main study (including methods and procedures of this sub-study) was approved by the ethical committee of the Friedrich-Alexander University Erlangen-Nürnberg (approval number: 210_17B) and registered in ClinicalTrials.gov (number: NCT03306069).

In the main study, participants were randomly assigned to an inactive control group (CON), only receiving nutritional counselling, or different exercise groups, performing specific types of interval training plus nutritional counseling. Random assignment was achieved using a computerized random number generator (MinimPy, GNU GPL v3), independently of the researchers who were involved in data collection. Prior to randomization, participants were stratified into groups according to the primary outcome VO_2max_ (< 20, 20–25, and > 25 mL/kg/min), age (< 45 and ≥ 45 years), gender (male/female) and BMI (< 35 and ≥ 35 kg/m^2^) to achieve a more balanced distribution of participants' main characteristics.

### Participants

Participants were recruited through local newspaper advertisements. Inclusion criteria for this sub-study were: age ≥ 18 years, obesity (BMI ≥ 30 kg/m^2^) plus at least two additional cardiometabolic abnormalities, including increased waist circumference (> 88 cm for females and > 102 cm for males), hypertension (≥ 130 mmHg and/or diastolic blood pressure ≥ 85 mmHg), dyslipidemia [triglycerides: ≥ 150 mg/dL; high-density lipoprotein cholesterol (HDL-C): < 40 mg/dL for males and < 50 mg/dL for females] and hyperglycemia (≥ 100 mg/dL) [30], current employment in a sedentary occupation (e.g. office work), and a self-reported sedentary lifestyle (i.e. no specific sports training and engaging in less than 30 min of moderate PA on 3 days/week as defined by the American College of Sports Medicine [31]). Exclusion criteria were: clinical diagnosis of heart disease, cancer, severe orthopaedic conditions or other major health problems that might preclude safe participation in exercise and pregnancy. Inclusion and exclusion criteria were discussed and clarified individually with each participant by the principle investigator at the first screening appointment before study enrolment. Participants agreed not to change their medications or dosages and to maintain their usual lifestyle patterns throughout the study to minimize potential confounding effects.

Based on previously published data from our group [29], suggesting a large effect (Cohen’s d = 0.97) on the primary outcome (relative VO_2max_), an a priori sample size calculation for the main study indicated that 16 participants per group would be required to yield a power of 0.95 in a 2-sided test with a 5% level of significance (G*Power, version 3.1.9.2). However, given that the literature reports attrition rates in obesity interventions of up to 80% [32], we expected considerably more dropouts than observed in normal-weight participants [29]. Thus, we aimed to recruit a minimum of 30 participants per group for the main study to sufficiently account for dropouts. In the present investigation, we used the data of participants from the main study, who were allocated to either low-volume HIIT or CON and who met the specific inclusion criteria of the sub-study.

**Fig. 1:**
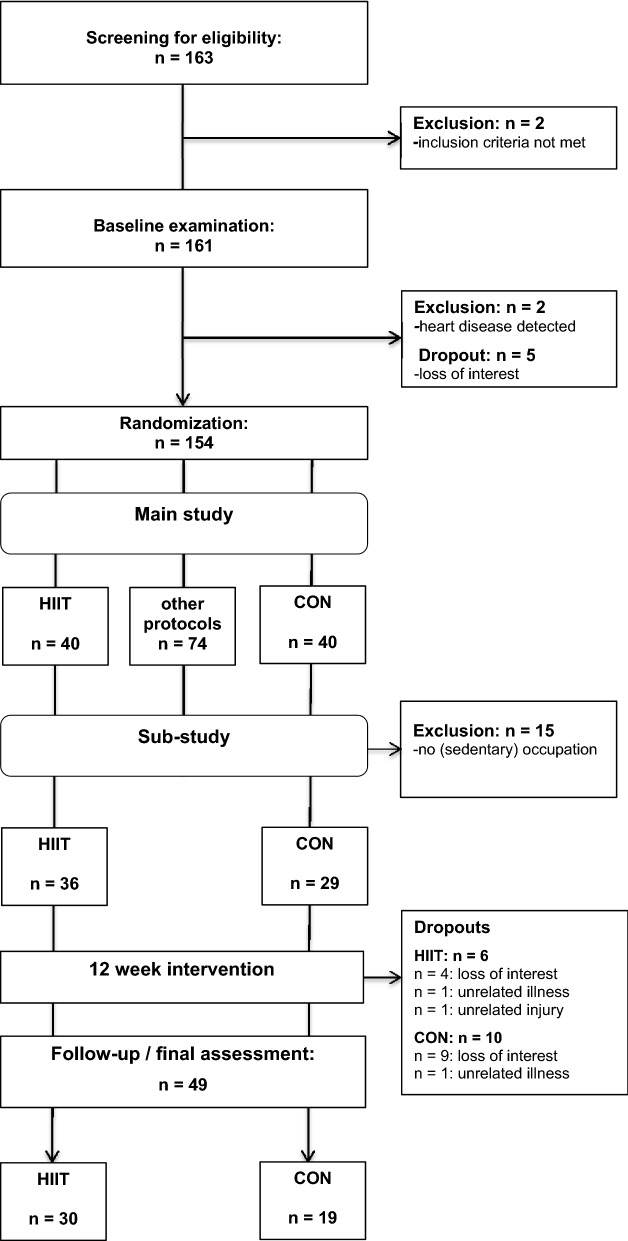
Study flow chart

A total of 163 individuals were screened for eligibility in the main study. Two participants were excluded for not meeting the inclusion criteria, 5 withdrew for personal reasons and 2 were excluded due to medical reasons detected during the health examination. Seventy-four participants were randomized to other interval training modalities not reported in this sub-study. Of 80 participants assigned to either HIIT (n = 40) or CON (n = 40), 15 participants were not included because they were unemployed or not employed in a predominately sedentary occupation. Of the remaining 65 participants, 16 dropped out during the intervention period (HIIT: n = 6; CON: n = 10). The reasons for dropout are displayed in Fig. 1 (Study Flow Chart). Thus, a total of 49 participants completed the study and were included in the final analysis (HIIT: n = 30; CON: n = 19). Participants’ main baseline characteristics and medication use are shown in Table 1.

**Table 1:** Baseline characteristics of study participants and medications

	HIIT (n = 36)	CON (n = 29)
Variable		
Men/women (n)	19/17	10/19
Age (years)	48.5 ± 10.0	49.0 ± 9.9
Body mass index (kg/m^2^)	40.4 ± 7.2	38.5 ± 6.9
VO_2max_ (mL/kg/min)	21.9 ± 6.2	21.7 ± 7.1
Medications, n (%)		
Beta blockers	2 (5.6%)	3 (10.3%)
ACE inhibitors	10 (27.8%)	8 (27.6%)
Non ACE inhibitors	7 (19.4%)	4 (13.8%)
Metformin	5 (13.9%)	3 (10.3%)
Exogenous insulin	1 (2.8%)	0
Anticoagulants	1 (2.8%)	0
Bronchodilators	1 (2.8%)	1 (3.4%)
Antihistamines	0	3 (10.3%)
l-thyroxine	4 (11.1%)	8 (27.6%)
Analgesics	8 (22.2%)	9 (31.0%)
Anti-depressants	5 (13.9%)	4 (13.8%)

*HIIT* High-intensity interval training group, *CON* control group

### Health examination

All procedures were carried out under laboratory conditions in a stable ambient environment and were strictly standardized as outlined below. Baseline examinations were carried out 1 week before the start of the intervention. Outcome reassessment was conducted within the first week after completion of the intervention at least 3 days apart from the last training session and at a similar time each day to ensure sufficient recovery and to avoid possible circadian effects. Participants were instructed to arrive overnight-fasted at our Research Center and to refrain from alcohol and vigorous PA for at least 24 h prior to both the baseline and follow-up examination. The assessments were made in a single-blinded fashion, meaning that the personnel who collected the data were not aware of the participants’ group assignment.

### Blood pressure measurements

After arrival at the laboratory, participants were initially asked to empty their bladder before the measurements were conducted. Subsequently, participants rested in a seated position for 5 min, and then systolic and diastolic blood pressure values were measured using an automatic upper-arm blood pressure monitor (M5 professional, Omron, Mannheim, Germany), which has been validated for accuracy [33]. Two consecutive measurements on both arms were obtained at 60-s intervals and their averaged values of the arm with the higher pressure were used in the analysis as recommended by the American College of Cardiology [34].

### Blood sampling

Blood samples were drawn via venipuncture from an antecubital arm vein into collection tubes using a disposable cannula (S-Monovette®, Sarstedt, Nürmbrecht, Germany). All blood samples were analyzed by the diagnostic laboratories of the University Hospital Erlangen. Serum values of glucose, triglycerides, total-cholesterol, low-density lipoprotein cholesterol (LDL-C) and high-density lipoprotein cholesterol (HDL-C) were measured photometrically (Clinical Chemistry Analyzer AU700 or AU5800, Beckman Coulter, Brea, CA, USA). The mean coefficients of variation (CV) for these analytes ranged between 1.1 and 1.4%. Serum glycosylated hemoglobin A_1c_ (HbA_1c_) was determined using a turbidimetric immuneassay (COBAS Integra 400, Roche Diagnostics, Mannheim, Germany, CV: 2.7%).

### Anthropometric and body composition measurements

Waist circumference was measured with a measuring tape while participants were in a standing position. Body composition measurements were conducted using a segmental multi-frequency bioelectrical impedance analysis device (seca mBCA 515, Seca, Hamburg, Germany), which has been shown to provide accurate determination of body composition in obese individuals when compared to the 4-compartement reference method [35]. All measurements were performed according to the manufacturer’s instructions.

### Cycle ergometer test

Participants performed a standardized ramp exercise test on an electronically braked cycle ergometer (Corival cpet, Lode, Groningen, Netherlands) to determine VO_2max_, maximal power output (W_max_) and maximal heart rate (HR_max_). Following a 1 min familiarization period, the initial load was set at 50 W and then gradually increased by 1 W every 5 s (i.e. 25 W within 2 min) in female participants and by 1 W every 4 s (i.e. 30 W within 2 min) in male participants, respectively, until volitional exhaustion. HR was recorded continuously using a 12-lead ECG system (custo cardio 110, custo med, Ottobrunn, Germany). VO_2max_ was measured with an open-circuit breath-by-breath spiroergometric system (Metalyzer 3B-R3, Cortex Biophysik, Leipzig, Germany), which has been shown to be a reliable instrument for cardiopulmonary exercise testing [36]. Before each test, the device was calibrated according to the manufacturer’s instructions. Criteria to assume that maximal effort was reached were at least two of the following: a leveling-off of oxygen uptake, maximal respiratory exchange ratio (RER_max_) ≥ 1.10, ≥ 90% of age predicted HR_max_ (APHR_max_, using the equation: 220–age) and maximal rate of perceived exertion (RPE_max_) ≥ 19 (assessed at exhaustion using the 6–20 Borg scale [37]) [38, 39]. In addition, the ventilatory threshold (VT) was determined according to the V-slope method by plotting carbon dioxide output (VCO_2_) against oxygen uptake (VO_2_) in order to assess submaximal exercise capacity [40].

### Assessment of self-reported measures

Self-reported outcomes were assessed using standardized questionnaires, which were all previously validated in the German language. Participants’ subjective work ability was examined using the Work Ability Index (WAI), which covers different dimensions including individual health, skills, and work environment. The score ranges from 7 to 49 with higher values indicating higher perceived work ability [41]. The EQ-5D-5L questionnaire was used to assess health-related QoL. The questionnaire consists of a visual analogue scale (EQ-VAS, 0–100 points, higher values indicate higher QoL) and a descriptive system of 5 health-related QoL-dimensions (mobility, self-care, usual activities, pain/discomfort, anxiety/depression) with 5 severity levels each, which are converted to a single index value (EQ-5D-5L). An index value of 1.0 represents the best possible state of perceived health, while an index value of 0 represents the worst possible health status [42]. The Perceived Stress Questionnaire (PSQ) was applied to examine the perception of stress, including the 4 sub-factors “worries”, “tension”, “joy”, and “demands”. Higher values (except for “joy”) indicate higher perceived stress [43]. Additionally, participants provided a personal evaluation sheet, including their enjoyment of the intervention on a 7-point rating scale (1 = not enjoyable at all; 7 = extremely enjoyable).

### Nutritional counseling

Participants received nutritional counseling by a registered dietitian in a face-to-face meeting. Dietary advices to promote weight loss were given in accordance to international guidelines for the treatment of obesity [44]. Nutritional intake was monitored by 24 h-dietary records (Freiburger Ernährungsprotokoll; Nutri-Science, Freiburg, Germany) assessed on 3 consecutive days at study entry and within the last week of the intervention. Computer-based analysis of mean caloric and nutrient intake was done by the software Prodi^®^6 expert (Nutri-Science, Freiburg, Germany).

### High-intensity interval training (HIIT)

Exercise sessions were performed on electronically braked cycle ergometers (Corival cpet, Lode, Groningen, Netherlands) at our Research Center and supervised by certified physiotherapists/sports therapists, who were trained in implementing the specific HIIT protocol.

The HIIT protocol was similar to the protocol developed by Reljic et al. [29]. Specifically, the protocol consisted of a 2 min warm-up phase, 5 interval bouts of 1 min at 80–95% HR_max_ interspersed with 1 min of low intensity recovery and a 3 min cool-down phase (total session time: 14 min). To reach their individual target HR for each interval bout, participants were advised to adjust the pedal cadence and/or increase load resistance and received verbal encouragement, if necessary, to ensure compliance with the protocol. Participants were provided with a chest strap HR monitor (Polar H7 heart rate sensor, Polar Electro Oy, Kempele, Finland) to continuously track their HR values during exercise and with individual training cards, where the target HR values were noted. Participants’ HR values were recorded throughout each exercise session and subsequently, HR responses during each interval were analyzed using a specific HR monitoring system (Polar Team, Polar Electro Oy, Kempele, Finland). The HIIT sessions were conducted twice a week (with at least 1 day rest in between) over a period of 12 weeks. Participants were able to schedule their exercise sessions individually during the opening hours of the training center.

### Statistical analysis

All statistical analyses were performed using SPSS version 24.0 (SPSS Inc., Chicago, IL, USA). First, the distribution of data was checked using the Shapiro–Wilk test. A 2 × 2 repeated-measures ANOVA was conducted to test for main effects of group (HIIT vs. CON), time (pre- vs. post-intervention) and interaction between both factors. Homogeneity of variance was verified with the Levene’s test. When significant main or interaction effects were found, post hoc paired t-tests were performed to determine within-group differences between pre- and post-intervention values and independent t-tests to assess between-group differences. In case of non-normally distributed data, log-transformation was used and the same analyses were applied to the transformed values. If log-transformation did not lead to data normalization (self-reported outcomes), the non-parametric Friedman two-way analysis of variance by ranks was conducted, followed by Wilcoxon’s and Mann–Whitney tests for post-hoc comparisons. Effect sizes were calculated using the partial eta-squared (ɳ_p_^2^) for ANOVA, Kendall’s coefficient of concordance (W) for the Friedman test and Cohen’s d for post hoc tests. Pearson (*r*) or Spearman (ρ) correlation analyses were calculated to investigate the relationship between selected parameters. For all analyses, the significance level was set at P < 0.05. Data are reported as means ± standard deviation (SD) and pre-/post-intervention changes are presented with 95% confidence intervals (95% CI), where appropriate.

## Results

### Anthropometric data and body composition

ANOVA showed significant main time effects for body weight (P < 0.001, ή^2^ = 0.50), BMI (P < 0.001, ή^2^ = 0.50), fat mass (P < 0.001, ή^2^ = 0.41), percentage of body fat (P < 0.001, ή^2^ = 0.32), fat free mass (P = 0.005, ή^2^ = 0.16) and body water (P = 0.001, ή^2^ = 0.21). Post hoc analyses revealed that both groups reduced body weight significantly after the intervention [HIIT: − 5.3 kg (95% CI − 7.3 to − 3.3 kg), P < 0.001, d = − 1.00; CON: − 3.7 kg (95% CI − 5.2 to − 2.1 kg), P < 0.001, d = − 1.33], mainly due to a reduction of body fat mass [HIIT: − 4.7 kg (95% CI − 6.6 to − 2.8 kg), P < 0.001, d = − 0.92; CON: − 2.8 kg (95% CI − 5.2 to − 2.1 kg), P = 0.001, d = − 1.06], and, to a smaller extent, a loss of body water [HIIT: − 0.4 L (95% CI − 1.0 to − 0.1 L), P = 0.02, d = − 0.38; CON: − 0.8 L (95% CI − 1.5 to − 0.1 L), P = 0.02, d = − 0.57]. In both groups, post hoc tests did not reveal significant changes in fat free mass. Moreover, a significant group-by-time interaction (P = 0.001, ή^2^ = 0.23) and main time effect (P < 0.001, ή^2^ = 0.40) was found for waist circumference. Post hoc tests showed only a significant decrease of waist circumference in the HIIT group by [− 7.5 cm (95% CI − 9.8 to − 5.1 cm), P < 0.001, d = − 1.18] (Table 2).

**Table 2:** Anthropometric and body composition data before and after the intervention

Variable	HIIT group (n = 30)		Control group (n = 19)		ANOVA P-value		
	Baseline	Post	Baseline	Post	Time	Group	Group × time
Body weight (kg)	121.9 ± 28.9	116.6 ± 28.1^***^	109.4 ± 18.3	105.7 ± 19.7^***^	< 0.001	0.147	0.501
Body mass index (kg/m^2^)	39.9 ± 7.5	38.1 ± 7.3^***^	37.5 ± 5.9	36.2 ± 6.2^***^	< 0.001	0.298	0.298
Fat mass (kg)	53.6 ± 17.3	48.9 ± 16.5^***^	48.4 ± 12.4	45.7 ± 14.1^**^	< 0.001	0.360	0.142
Fat mass (%)	43.7 ± 7.7	41.7 ± 8.0^***^	44.3 ± 8.7	42.9 ± 9.3^**^	< 0.001	0.807	0.424
Fat free mass (kg)	68.3 ± 16.9	67.7 ± 17.1	60.9 ± 13.7	60.1 ± 13.9	0.005	0.110	0.509
Body water (L)	50.9 ± 12.3	50.4 ± 12.5^*^	45.7 ± 10.0	44.9 ± 9.5^*^	0.001	0.116	0.496
Waist circumference (cm)	120.5 ± 18.9	113.0 ± 18.0^***^	110.7 ± 11.1	109.2 ± 11.8	< 0.001	0.154	0.001

*HIIT* High-intensity interval training group, *CON* control group

* P < 0.05, ** P < 0.01, *** P < 0.001 significant within-group difference between baseline and post-intervention

### Nutritional analysis

Four participants [2 from each group] missed to provide a complete follow-up dietary record and were thus not included in the nutritional evaluation. Total energy and macronutrient intake was comparable between both groups. Nutritional analyses revealed that the average daily energy intake decreased in both groups [HIIT: − 346 kcal (95% CI − 733 to 342 kcal); CON: − 219 kcal (95% CI − 923 to 90 kcal)], although these changes did not reach statistical significance. In both groups, caloric restriction was mainly achieved through a reduction in carbohydrate intake. There was a significant main effect of group for total protein intake (P = 0.018, ή^2^ = 0.12). Post hoc test showed that protein intake tended to be higher in the HIIT group compared to CON at follow-up but the difference between groups did not reach statistical significance (P = 0.050). In both groups, the changes in macronutrient intakes were not statistically significant between the two time points (Table 3).

**Table 3:** Daily nutritional intake before and during the last week of the intervention

Variable	HIIT (n = 28)		Control group (n = 17)	
	Baseline	Post	Baseline	Post
Total energy (kcal)	2529 ± 1136	2183 ± 917	2066 ± 714	1847 ± 846
Protein (g)	122 ± 84	110 ± 55	85 ± 30	83 ± 34
Protein (% total kcal)	18 ± 6	22 ± 11	17 ± 3	20 ± 6
Fat (g)	100 ± 42	90 ± 45	82 ± 36	74 ± 47
Fat (% total kcal)	39 ± 12	37 ± 8	35 ± 7	34 ± 7
CHO (g)	241 ± 112	208 ± 105	217 ± 68	182 ± 74
CHO (% total kcal)	42 ± 14	38 ± 10	44 ± 8	42 ± 9
Fiber (g)	22 ± 11	22 ± 12	22 ± 12	24 ± 11
Fiber (% total kcal)	2 ± 1	2 ± 1	2 ± 1	3 ± 1

*HIIT* High-intensity interval training group, *CON* control group, *CHO* carbohydrates

### Cardiorespiratory fitness and physical performance

The average baseline VO_2max_ (21.8 ± 6.5 mL/kg/min) indicated that the CRF level was generally very poor in the total sample. One participant in the CON group was not able to perform the post-intervention cycle ergometer test due to an injury unrelated to the study.

Pre- and post-intervention, all participants reached at least two maximal effort criteria during the cycle ergometer test. Plateau in VO_2_ was reached by 100%, RER_max_ ≥ 1.10 by 13% and HR_max_ ≥ 90% of APHR_max_ by 75% of participants, respectively, at both time points. RPE_max_ ≥ 19 was reached by 100% of participants pre-intervention and by 98% post-intervention, respectively. ANOVA revealed a significant group-by-time interaction for HR_max_ (P = 0.003, ή^2^ = 0.17) and %APHR_max_ (P = 0.004, ή^2^ = 0.17). Post hoc tests showed significantly lower HR_max_ [− 5 beats/min (95% CI − 8 to − 3 beats/min), P < 0.001, d = − 1.14] and %APHR_max_ [− 3% (95% CI − 5 to − 2%), P < 0.001, d = − 1.02] values at the post-intervention cycle ergometer test in the CON group. All other maximal effort data were not significantly different between baseline and follow-up in both groups, suggesting that a similar level of exertion was achieved at both time points (Additional file 1).

A significant group-by-time interaction and main effect of time was observed for relative VO_2max_ (P < 0.001, ή^2^ = 0.40 and P < 0.001, ή^2^ = 0.25, respectively), absolute W_max_ (P < 0.001, ή^2^ = 0.61 and P < 0.001, ή^2^ = 0.30, respectively), relative W_max_ (P < 0.001, ή^2^ = 0.38 and P < 0.001, ή^2^ = 0.40, respectively) and VT-performance (P < 0.001, ή^2^ = 0.52 and P < 0.001, ή^2^ = 0.49, respectively). Moreover, a significant group-by-time interaction was seen for absolute VO_2max_ (P < 0.001, ή^2^ = 0.40). HIIT improved absolute VO_2max_ (+ 270 mL [95% CI 180 to 367 mL], P < 0.001, d = 1.15), relative VO_2max_ (+ 3.5 mL//kg/min [95% CI 2.5 to 4.6 mL/kg/min], P < 0.001, d = 1.24), absolute W_max_ (+ 24 W [95% CI 19 to 30 W], P < 0.001, d = 1.73), relative W_max_ (+ 0.3 W/kg [95% CI 0.2 to 0.4 W/kg], P < 0.001, d = 1.59) and VT-performance (+ 32 W [95% CI 26 to 39 W], P < 0.001, d = 2.00) significantly after the intervention. In CON, absolute VO_2max_ [− 170 mL (95% CI − 271 to − 69 mL), P = 0.002, d = − 1.04] and absolute W_max_ [− 8 W (95% CI − 13 to − 2 W), P = 0.011, d = − 0.61] were significantly decreased after the intervention. Post-intervention absolute VO_2max_ and VT-performance were significantly higher in the HIIT group compared to CON by 643 mL [(95% CI 166 to 1021 mL), P = 0.009, d = 0.95] and 22 W [(95% CI 3 to 40 W), P = 0.025, d = 0.72], respectively (Table 4).

**Table 4:** Cardiorespiratory fitness and exercise performance variables before and after the intervention

Variable	HIIT group (n = 30)		Control group (n = 18)		ANOVA P-value		
	Baseline	Post	Baseline	Post	Time	Group	Group × Time
VO_2max_ (L/min)	2.7 ± 0.8	3.0 ± 0.7^***++^	2.5 ± 0.8	2.3 ± 0.8^**^	0.146	0.080	< 0.001
VO_2max_ (mL/kg/min)	22.5 ± 6.5	26.0 ± 6.6^***^	23.1 ± 8.0	22.5 ± 8.7	< 0.001	0.486	< 0.001
W_max_ (W)	168 ± 51	192 ± 48^***^	171 ± 64	164 ± 63^*^	< 0.001	0.444	< 0.001
W_max_ (W/kg)	1.4 ± 0.5	1.7 ± 0.5^***^	1.6 ± 0.6	1.6 ± 0.7	< 0.001	0.842	< 0.001
HR_max_ (b/min)	162 ± 15	164 ± 15	163 ± 23	157 ± 22^***^	0.309	0.593	0.003
Power at VT (W)	63 ± 28	96 ± 30^***+^	75 ± 32	74 ± 32	< 0.001	0.580	< 0.001

*HIIT* High-intensity interval training group, *CON* control group, *VO*_*2max*_ maximal oxygen uptake, *W*_*max*_ maximal power output in watts, *HR*_*max*_ maximal heart rate, *VT* ventilatory threshold

* P < 0.05, ** P < 0.01, *** P < 0.001 significant within-group difference between baseline and post-intervention

^+^P < 0.05, ^++ ^P < 0.01 significant difference between groups

### Cardiometabolic risk markers

ANOVA showed a significant group-by-time interaction and main effect of time for systolic blood pressure (P = 0.006, ή^2^ = 0.15 and P = 0.001, ή^2^ = 0.23, respectively), diastolic blood pressure (P = 0.009, ή^2^ = 0.14 and P < 0.001, ή^2^ = 0.39, respectively) and mean arterial blood pressure (P = 0.002, ή^2^ = 0.19 and P < 0.001, ή^2^ = 0.39, respectively). Post hoc tests demonstrated that systolic blood pressure [− 12 mmHg (95% CI − 16 to − 8 mmHg), P < 0.001, d = − 1.04], diastolic blood pressure [− 10 mmHg (95% CI − 13 to − 7 mmHg), P < 0.001, d = − 1.17] and mean arterial blood pressure [− 11 mmHg (95% CI − 14 to − 8 mmHg), P < 0.001, d = − 1.39] values were significantly decreased in the HIIT group. No significant changes in blood pressure values were observed in the CON group. Moreover, ANOVA showed a significant main effect of time for HbA_1c_ (P = 0.045, ή^2^ = 0.08) and a significant main effect of group for HDL-C (P = 0.033, ή^2^ = 0.09). However, subsequent post hoc tests did not reveal any significant within- or between-group differences (Table 5).

**Table 5:** Cardiometabolic risk variables before and after the intervention

Variable	HIIT group (n = 30)		Control group (n = 19)		ANOVA P-value		
	Baseline	Post	Baseline	Post	Time	Group	Group × time
Systolic BP (mmHg)	147 ± 17	135 ± 15^***^	137 ± 11	136 ± 12	0.001	0.259	0.006
Diastolic BP (mmHg)	96 ± 10	86 ± 10^***^	90 ± 9	87 ± 10	< 0.001	0.378	0.009
MAB (mmHg)	113 ± 11	102 ± 11^***^	106 ± 9	103 ± 10	< 0.001	0.281	0.002
Glucose (mg/dL)	101 ± 18	102 ± 12	99 ± 18	95 ± 16	0.345	0.842	0.242
HbA_1c_ (%)	5.6 ± 0.4	5.5 ± 0.4	5.5 ± 0.4	5.4 ± 0.4	0.045	0.121	0.648
Triglycerides (mg/dL)	137 ± 57	131 ± 49	134 ± 67	124 ± 57	0.330	0.578	0.696
Cholesterol (mg/dL)	219 ± 36	213 ± 36	223 ± 48	215 ± 42	0.124	0.783	0.780
HDL-C (mg/dL)	47 ± 10	48 ± 11	55 ± 14	55 ± 14	0.973	0.033	0.605
LDL-C (mg/dL)	148 ± 30	141 ± 30	145 ± 38	141 ± 33	0.157	0.756	0.863
LDL/HDL ratio	3.2 ± 0.9	3.0 ± 0.8	2.8 ± 0.8	2.7 ± 0.7	0.092	0.058	0.521

*HIIT* High-intensity interval training group, *CON* control group, *BP* blood pressure, *MAB* mean arterial blood pressure*, HbA*_*1c*_ glycosylated hemoglobin A_1c_, *HDL-C* high-density lipoprotein cholesterol, *LDL-C* low-density lipoprotein cholesterol

* P < 0.05, *** P < 0.001 significant within-group difference between baseline and post-intervention

### Self-reported variables

Friedman tests revealed significant changes in WAI (P = 0.018, *W* = 0.12), EQ-5D-5L (P = 0.048, *W* = 0.08) and EQ-VAS (P < 0.001, *W* = 0.28) over time. Group-specific analyses showed significant improvements in WAI (+ 3.0 points, P < 0.001, d = 1.90), EQ-5D-5L (+ 0.04 points, P = 0.033, d = 0.85) and EQ-VAS (+ 10%, P < 0.001, d = 1.93) in the HIIT group. No significant changes in self-reported outcomes were observed in the CON group. Post-intervention WAI scores were significantly higher in the HIIT group compared to CON (+ 2.7 points, P = 0.046, d = 0.67) (Table 6). There were significant positive correlations between relative VO_2max_ and WAI (ρ = 0.32, P = 0.012), EQ-5D-5L (ρ = 0.33, P = 0.009), EQ-VAS (ρ = 0.39, P = 0.003), and the perception of joy (ρ = 0.31, P = 0.016). Increases in relative VO_2max_ were significantly correlated with increases in WAI (ρ = 0.44, P = 0.001), and decreases in the perception of worries (ρ = 0.42, P = 0.002). Improvements in VT-performance were significantly correlated with increases in WAI (ρ = 0.40, P = 0.002) and tended to be associated with increases in EQ-VAS (ρ = 0.24, P = 0.053). Changes in body weight were not significantly correlated with changes in participant-based outcomes.

**Table 6:** Self-reported variables before and after the intervention

Variable	HIIT group (n = 30)		Control group (n = 19)		Friedman test P-value
	Baseline	Post	Baseline	Post	
Work ability Index	34.6 ± 6.8	37.6 ± 7.3^***+^	36.9 ± 4.7	34.9 ± 6.5	0.018
EQ-5D-5L (index)	0.87 ± 0.14	0.90 ± 0.15^*^	0.88 ± 0.12	0.85 ± 0.21	0.048
EQ (VAS)	67 ± 14	77 ± 17^***^	63 ± 22	67 ± 27	< 0.001
PSQ-worries	33.8 ± 24.1	27.8 ± 23.0	25.6 ± 17.9	24.8 ± 15.6	0.206
PSQ-tension	45.8 ± 26.9	38.2 ± 24.8	37.9 ± 27.9	38.2 ± 26.4	0.160
PSQ-joy	55.8 ± 24.3	63.1 ± 24.0	62.5 ± 25.7	61.1 ± 23.1	0.078
PSQ-demands	45.2 ± 22.8	47.6 ± 21.9	41.8 ± 23.8	37.2 ± 23.2	0.631
PSQ-total	42.3 ± 21.3	37.6 ± 20.3	35.7 ± 20.6	34.9 ± 18.7	0.189

*HIIT* High-intensity interval training group, *CON* control group; *VAS* visual analogue scale, *PSQ* Perceived Stress Questionnaire

* P < 0.05, *** P < 0.001 significant within-group difference between baseline and post-intervention

^+^P < 0.05 significant difference between groups

### Safety and acceptability

No adverse events occurred at any time point during the training sessions. The average peak HR reached at the end of each interval bout was equivalent to 94.5 ± 4.0% of HR_max_, confirming that the prescribed level of exercise intensity was reached in the HIIT group. The adherence rate (the percentage of the scheduled training sessions that the participants completed) in the HIIT group was 94.3 ± 7.9%. The average enjoyment of the exercise protocol was rated with 6.0 ± 0.9 points on a 7-point rating scale.

The most common reasons mentioned by participants for insufficient PA prior to the study were “lack of time” and “poor motivation” (50% each), followed by “physical complaints” (20%), and “weight stigma” (16%). 80% of participants in the HIIT group stated that the applied HIIT protocol was helpful to overcome previously perceived barriers to regular exercise and 90% stated that they intended to further engage regularly in HIIT after termination of the study.

## Discussion

The major findings of this study were that: (i) low-volume HIIT led to significant improvements in cardiometabolic health, work ability and well-being in severely obese individuals after only 12 weeks, and (ii) caloric restriction was helpful for the reduction of body weight, however, only participants who additionally exercised experienced profound positive changes in physical and psychological health outcomes.

Several studies have shown that HIIT can elicit various physiological adaptations that are linked to improved health outcomes [18–20]. However, to date, data on the feasibility and efficacy of low-volume HIIT applied to severely obese individuals at increased cardiometabolic risk are sparse. The average weight loss achieved in our study (~ 4%) was slightly higher than the mean value (3%) reported in most obesity programs [45]. Although a weight loss of at least 5% has been suggested as clinically meaningful and a criterion to define a “successful” obesity treatment, respectively, it has been reported that even lesser amounts of weight loss may provide beneficial health effects [46, 47].

Moreover, there is strong evidence that increasing PA is associated with a decrease in cardiometabolic risk despite little or no change in body weight [47] and that CRF is a more powerful predictor of morbidity and mortality than BMI or body fat distribution [48, 49]. It is well established that a low VO_2max_ is a key predictor for CVD and mortality. Estimates indicate that an increase in relative VO_2max_ by 3.5 mL/kg/min is associated with a 10–25% reduction in all-cause mortality risk [10]. Thus, the observed increase in VO_2max_ from 22.5 ± 6.5 to 26.0 ± 6.6 ml/kg/min (~ 16%) in our participants can be considered highly clinically significant. An additional intention-to-treat analysis, which was performed using data from all initial 65 participants (including dropouts) showed similar results and thus strengthens our finding that low-volume HIIT may elicit profound improvements in CRF in severely obese individuals at increased cardiometabolic risk (Additional file 2).

To date, there have been only a few studies with relatively small sample sizes investigating the effects of HIIT in severely-to-morbidly obese individuals. Lanzi et al. [50] observed a ~ 12% increase in VO_2max_ after 8 sessions of HIIT combined with an adapted diet in 9 men with class II and III obesity. More recently, Clark et al. [51] reported a 4–5% increase in VO_2max_ following 6 weeks of HIIT in 17 women with an average BMI of 39.1 kg/m^2^. The greater increase in VO_2max_ observed in our study may be attributed to the longer intervention period and/or due to differences in the applied HIIT protocol. The average intensity reached during the interval bouts in our study was equivalent to ~ 95% HR_max_, which was higher than the reported values in the two previous studies (~ 90% HR_max_). It is to note that the time effort for our protocol (14 min/session) was ~ 50% lower than in the former studies with session durations ranging from 25–30 min, which may be an important factor for longer-term adherence. In accordance with recent research [29, 52–54], these findings suggest that exercise intensity appears to be the more critical factor for improving CRF than exercise volume.

We additionally assessed power output at VT, a submaximal marker of CRF, which is more specific to determine the ability to perform physical activities of daily living [40]. The significant increase in VT-performance observed in our study may indicate metabolic adaptations (e.g. mitochondrial biogenesis in the skeletal muscle) in response to HIIT [19] that are linked with enhanced capacity to perform sustained submaximal activities.

The deterioration of VO_2max_ in CON group participants, who achieved weight loss through caloric restriction alone, is in line with previous research. Weiss et al. [55], for example, reported a 6% decrease in aerobic capacity in overweight women, who lost 7% of body weight during 16 weeks of 20% calorie reduction. By contrast, a comparative group in this study, who reduced a similar amount of body weight through 10% calorie reduction and 10% increased energy expenditure through moderate/vigorous PA was able to maintain CRF. The underlying physiological mechanisms responsible for the deterioration of CRF after weight loss from caloric restriction are not yet fully understood but may be related to skeletal muscle atrophy, catabolic processes in the cardiovascular system [55] and unfavorable changes in endocrine and haemotopoietic systems [56].

As well-established cardiometabolic risk markers, excess abdominal fat and high blood pressure are associated with CVD and mortality [57, 58]. It has been reported that a 10% reduction in waist circumference (as surrogate marker for abdominal fat) corresponds to a ~ 1.5 times lower mortality risk [57] and that every 10 mmHg reduction in systolic blood pressure lowers risk of CVD and mortality by 20% and 13%, respectively [58]. The observed reductions in waist circumference (~ 6%) and systolic blood pressure (~ 12 mmHg) in the HIIT group are therefore very likely to provide clinically relevant benefits, comparable to effects obtained in pharmacological studies [58]. It has to be emphasized that the reduction in waist circumference was significantly greater in the HIIT group, despite similar changes in body weight between both groups. This finding is consistent with previous studies reporting exercise-induced reductions in waist circumference despite minimal or lack of body weight loss [47]. In this context, it has been suggested that parallel but opposing changes in body fat mass and lean mass may occur in response to increased PA that cannot be detected by body weight changes but by waist circumference and body composition, respectively [47]. In accordance with these previous reports, our findings once again underline that body weight reduction should not be regarded as the only or key indicator of a successful obesity treatment, and support the recommendation that body composition and waist circumference should be a routine measure to identify cardiometabolic risk in obese individuals [47].

Compared to a number of previous studies reporting beneficial effects of HIIT on fasting glucose [19, 20], total cholesterol [21], LDL-C [21] and HDL-C [59], we found no significant changes in blood markers of glucose and lipid metabolism. It might be speculated, therefore, that the total energy expenditure from our extremely low-volume HIIT protocol, which was performed with a frequency of only twice a week, may have been too low to induce positive alterations in participants’ glycemic and lipid profiles. In accordance with this assumption, a recent meta-analysis has reported that HIIT protocols consisting of interval durations of ≥ 2 min appear to have a greater impact on energy expenditure and related physiological adaptions than protocols with shorter exercise bouts [21]. However, in contrast, we have previously observed significant reductions in LDL-C levels following 8 weeks of our low-volume HIIT in normal-weight participants [29]. Moreover, two other studies applying brief “all-out” sprint interval training protocols in sedentary individuals [54] and type 2 diabetes patients [60] observed positive glycemic effects despite a substantially lower exercise volume and time commitment than traditional MICT. Since most of the previous studies have included normal-weight, overweight and moderately obese individuals, it might be conceivable that the insufficient improvements in blood lipid profiles and glycemic control observed in the present study may be due to pre-existing less favorable metabolic conditions in our severely obese participants. Thus, further studies in these populations are needed to draw more comprehensive conclusions on this issue.

Although not the subject of the current study, it was a noteworthy finding that only a small proportion of participants, both in the HIIT and CON group, reached RER_max_ ≥ 1.10 (which is typically suggested as a cut-off value indicating that maximal effort has been achieved) at exhaustion during the cycle ergometer tests, despite reaching at least two other maximal effort criteria [38, 39]. In accordance with other reports [61, 62], this finding may be due to altered substrate utilization during exercise in obese individuals and supports previous studies indicating that the use of secondary criteria like RER_max_ to establish VO_2max_ may possibly be associated with errors under certain circumstances [39, 63].

Apart from physiological benefits related to PA, regular exercise is associated with a number of positive effects on psychological outcomes [64]. According to a recent meta-analysis, however, the effectiveness of structured exercise programs on work ability is still inconclusive, as only two out of six randomized controlled studies showed positive effects [65]. To our knowledge, this is the first study to investigate the effects of HIIT on work ability, QoL and perceived stress in severely obese sedentary employees. Baseline assessments revealed that on average, participants had a moderate WAI (~ 35 points) with need for improvement [41], and EQ-VAS scores (~ 65%), that were lower compared to normative values in the general population [42]. Our results indicate that low-volume HIIT may positively affect work ability and well-being in severely obese individuals as became evident by significant improvements in WAI and QoL.

Since obesity has been found to be associated with diminished QoL [6], lower work ability [9], and higher costs in the workplace [7], our low-volume HIIT protocol may be an interesting option for worksite health professionals interested in implementing feasible, time-efficient and effective exercise programs in overweight/obese employees. Given that cost-effectiveness plays an important role for decision-makers to grant worksite health-promotion programs [66], such extremely time-efficient exercise strategies may be a viable approach to increase companies’ willingness to support PA interventions.

Although HIIT-based exercise programs have recently gained increasing popularity, critics typically question whether untrained individuals would be physically able or willing to participate in vigorous exercise and argue that HIIT might be unsafe for obese people [67]. Hence, it is important to note that no adverse events occurred during the present study, suggesting that our low-volume HIIT protocol may be safely administered in severely obese individuals with pre-existing cardiometabolic disorders. Notwithstanding, as generally recommended before beginning an exercise program, it is especially important for individuals at increased cardiometabolic risk to first undergo a proper pre-participation health screening prior to engaging in HIIT. The high adherence rate (~ 94%) and the fairly low number of dropouts (~ 17%) when compared to other obesity interventions [32] indicate a good tolerability and high level of acceptance of HIIT among obese individuals. Given that higher exercise volume has been shown to be associated with higher dropout rates in HIIT interventions [68], it may be assumed that our time-efficient HIIT protocol could be a helpful approach to overcome time-related barriers to regular PA more easily.

Generally, the present HIIT protocol is not limited to cycle ergometers, but can also be applied using other modes of exercise. However, as the prevalence of physical complaints, such as joint problems, is typically increased with increasing body weight [69], we assume that HIIT is probably better tolerated by severely obese individuals when using non‐weight bearing exercise modes like cycle ergometers compared with higher‐impact exercises (e.g. treadmill). In line with this, a recent meta-analysis from our group revealed a higher prevalence of orthopedic complaints and significantly greater dropout rates in running/walking‐versus cycling‐based HIIT interventions with sedentary individuals [68]. We therefore suggest that exercise and health professionals planning to implement HIIT‐based programs for obese participants should rather focus on non‐weight bearing HIIT modalities to maximize tolerability.

There are some limitations of this study that should be considered. First, it is well-known that self-reported outcomes may be associated with potential sources off error, including dishonesty, conscientious responses or lack of memory. It cannot be ruled out, for example, that the positive effects on self-reported outcomes were biased by social desirability. Moreover, questionnaire-based assessments represent only a “snapshot” of how someone is feeling on a particular day and may not necessarily reflect a causal relationship. Nevertheless, previous research supports the positive impact of exercise on various psychological outcomes [64].

Second, non-probability sampling as used in this sub-study may be associated with limited generalization to the overall population, although we do not feel this had meaningful effects on the assessment of our outcomes. Third, albeit our study involved a longer intervention period and a larger sample size compared to previous HIIT studies in severely obese individuals, the long-term efficacy of low-volume HIIT and the long-term adherence to our protocol remain to be determined in obese populations. Larger-scale (ideally multicenter) studies involving long-term intervention periods will be needed to answer such questions.

Fourth, the mean age of our participants was ~ 50 years, which is not surprising in view of our inclusion criteria, since body weight and BMI typically reach peak values in this age group [70] and prevalence rates of cardiometabolic disorders have been found to be substantially higher than in younger ages [71]. However, as physiological adaptions to exercise may change with age, future studies may wish to investigate the effects of low-volume HIIT in younger and older obese populations. In particular, given that many industrialized countries worldwide are experiencing a shift in the age distribution of their populations, targeted exercise programs are becoming increasingly important for older adults and thus, further research will be important in ensuring the public health impact of low-volume HIIT.

Last, we note that the present study was conducted in a well-controlled setting with careful supervision of all exercise sessions. Therefore, further research will be needed to explore whether severely obese individuals would be able and/or willing to conform with the present low-volume HIIT protocol without a close supervision. Moreover, given that differences in modalities and delivery of the intervention may lead to different outcomes in “real‐world” conditions, future studies may wish to examine the feasibility of low-volume HIIT implemented directly in the workplace. Recent research, however, shows first promising evidence that low-volume HIIT could be feasibly applied to sedentary office workers in an unsupervised workplace setting [72].

## Conclusion

This study demonstrates that less than 30 min of low-volume HIIT per week may induce clinically relevant positive effects on cardiometabolic health, in particular VO_2max_, and significant improvements in work ability and well-being in severely obese individuals. Our findings highlight the crucial role of exercise in improving physical and psychological health that goes far beyond simple weight loss alone.

## Supplementary information

**Additional file 1.** Maximal effort data during the cycle ergometer test before and after the intervention.

**Additional file 2.** Intention-to-treat analysis of VO_2max_ before and after the intervention (including data of all participants who were enrolled in the study).

## Data Availability

The datasets generated and analyzed during the current study are not publicly available but are available from the corresponding author on reasonable request.

## Abbreviations

HIIT: High-intensity interval training
BMI: Body mass index
CON: Control group
VO_2max_: Maximal oxygen uptake
WAI: Work Ability Index
QoL: Quality of life
PSQ: Perceived Stress Questionnaire
CIs: Confidence intervals
CVD: Cardiovascular disease
PA: Physical activity
CRF: Cardiorespiratory fitness
SMD: Standardized mean difference
MICT: Moderate-intensity continuous training
HDL-C: High-density lipoprotein cholesterol
LDL-C: Low-density lipoprotein cholesterol
HbA_1c_: Glycosylated hemoglobin A1c
CV: Coefficient of variation
W: Watt
W_max_: Maximal power output
HR: Heart rate
HR_max_: Maximal heart rate
ECG: Electrocardiogram
RER_max_: Maximal respiratory exchange ratio
APHR_max_: Age predicted HR_max_
RPE_max_: Maximal rate of perceived exertion
VT: Ventilatory threshold
VCO_2_: Carbon dioxide output
VO_2_: Oxygen uptake
EQ-VAS: Health-related quality of life, visual analogue scale
EQ-5D-5L: Health-related quality of life, index value
SD: Standard deviation
r: Pearson’s correlation coefficient
ρ: Spearman’s rho
CHO: Carbohydrates
BP: Blood pressure
MAB: Mean arterial blood pressure
