# Supplementary material for: Low-volume high-intensity interval training improves cardiometabolic health, work ability and well-being in severely obese individuals: a randomized-controlled trial sub-study
Source: J Transl Med. 2020 Nov 7;18:419. doi: 10.1186/s12967-020-02592-6 (PMC7648946; doi:10.1186/s12967-020-02592-6)
Supplement: Supplementary file 1 — Additional file 1. Maximal effort data during the cycle ergometer test before and after the intervention. [file 12967_2020_2592_MOESM1_ESM.pdf]

Maximal effort data during the cycle ergometer test before and after the intervention

	HIIT group (n=30)		Control group (n=18)		ANOVA P-value		
**Variable**	**Baseline**	**Post**	**Baseline**	**Post**	**Time**	**Group**	**Group x Time**
VO_{2max} plateau (% of participants)	100%	100%	100%	100%	NA	NA	NA
%APHRmax (%)	95±10	97±9	95±12	92±12***	0.298	0.395	0.004
RER_{max} (VCO_2/VO_2)	1.04±0.1	1.04±0.1	1.02±0.1	1.02±0.1	0.351	0.200	0.889
RPE_{max} (15 point scale)	19.4±0.6	19.2±0.6	19.5±0.5	19.5±0.5	0.110	0.210	0.110

HIIT High-intensity interval training group, *CON* control group, VO_{2max} oxygen uptake, $APHR_{max}$ age-predicted maximal heart rate (220 – age), RER_{max} maximal respiratory exchange ratio, *RPE* rate of perceived exertion (Borg Scale 6-20); *NA* not applicable; ***(P<0.001) significant within-group difference between baseline and post-intervention
