# Supplementary material for: Low-volume high-intensity interval training improves cardiometabolic health, work ability and well-being in severely obese individuals: a randomized-controlled trial sub-study
Source: J Transl Med. 2020 Nov 7;18:419. doi: 10.1186/s12967-020-02592-6 (PMC7648946; doi:10.1186/s12967-020-02592-6)
Supplement: Supplementary file 2 — Additional file 2. Intention-to-treat analysis of VO2max before and after the intervention (including data of all participants who were enrolled in the study). [file 12967_2020_2592_MOESM2_ESM.pdf]

Intention-to-treat analysis of VO_{2max} before and after the intervention (including data of all participants who were enrolled in the study)

	HIIT group (n=36)		Control group (n=29)		ANOVA P-value		
**Variable**	**Baseline**	**Post**	**Baseline**	**Post**	**Time**	**Group**	**Group x Time**
VO_{2max} (L/min)	2.6±0.7	3.0±0.7^{***+++}	2.5±0.8	2.3±0.7*	0.186	0.017	<0.001
VO_{2max} (mL/kg/min)	21.9±6.2	26.0±6.0^{***+}	21.7±7.1	22.0±6.9	<0.001	0.218	<0.001

HIIT High-intensity interval training group, *CON* control group, *VO_{2max}* maximal oxygen uptake; *(P<0.05), ***(P<0.001) significant within-group difference between baseline and post-intervention; $^{+}$(P<0.05), $^{+++}$(P<0.001) significant difference between groups. Note: missing data of dropouts were handled with the imputation of the group mean.

1
